# Living Space Mobility and Liberty Deprivation Measures Among Residents With Dementia in Long‐Term Inpatient Care: A Longitudinal Study

**DOI:** 10.1002/nop2.70433

**Published:** 2026-01-20

**Authors:** Nico Marcus Haller, Lena Knüppel, Lars Kaderali, Simone Freitag, Steve Strupeit

**Affiliations:** ^1^ Institute of Nursing Science and Interprofessional Learning University Medicine Greifswald Greifswald Germany; ^2^ Institute of Bioinformatics University Medicine Greifswald Greifswald Germany

**Keywords:** dementia, liberty‐depriving measures, living space mobility, long‐term inpatient care, radius of movement

## Abstract

**Objectives:**

The aim of this study was to investigate the living space mobility among people with and without dementia and the influence of liberty‐depriving measures on living space mobility.

**Design:**

This empirical quantitative study used a longitudinal design.

**Methods:**

Residents in five inpatient long‐term care facilities were examined over a period of 6 months, including individuals with and without dementia. Data were collected at three time points between October 2023 and July 2024. Living space mobility was assessed by nursing staff using the Heidelberg Instrument for Assessing the Quality of Life of People with Dementia (H.I.L.D.E.), and the liberty deprivation measures were assessed by analysing medical records. The data were statistically analysed to obtain frequencies and to examine group differences.

**Results:**

There was a significant correlation between living space mobility and dementia diagnosis: residents with dementia mainly stayed within their living area, whereas residents without dementia had a greater range of movement, including outside the facility. A significant correlation between liberty deprivation measures and dementia diagnosis could not be established. However, living space mobility was significantly associated with the use of liberty deprivation measures regardless of dementia status.

**Conclusion:**

The results highlight the importance of living space mobility for residents with dementia and emphasise the influence of liberty deprivation measures. Further longitudinal studies with larger samples and alternative survey methods are necessary to investigate these relationships in greater depth.

**Implications for the Profession and/or Patient Care:**

For professional care, it is important to promote the mobility and well‐being of residents, regularly review freedom of movement and implement strategies for participation and autonomy in order to increase the quality of care.

**Impact:**

The results emphasise the importance of mobility promotion in dementia care and the critical reflection of liberty deprivation measures in long‐term inpatient care. Larger longitudinal studies are recommended to clarify the causal relationships.

**Reporting Method:**

The methods and results of this study were reported in accordance with the Strengthening the Reporting of Observational Studies in Epidemiology (STROBE) guidelines.

**Patient or Public Contribution:**

The study participants were recruited after being informed about the study and providing their consent.

## 1 Introduction

Dementia is a clinical syndrome that is characterised by a progressive deterioration of cognitive functions, ultimately resulting in the gradual loss of previously acquired skills and abilities (Federal Ministry of Health 2025). Globally, the prevalence of dementia is substantial; in 2019, over 55 million individuals were estimated to be living with the condition (World Health Organization 2021). It is estimated that this figure will nearly double within the next two decades, potentially reaching 139 million by 2050 (World Health Organization 2021).

In Germany, approximately 1.8 million individuals were affected by dementia at the end of 2023, with Alzheimer's disease, a subtype of dementia, constituting the predominant aetiology. In the same year, approximately 445.000 new cases of dementia were identified among individuals aged 65 years and older. The ongoing ageing of the global population is expected to further increase the prevalence of dementia in the coming years (German Alzheimer Society e.V. 2024).

This demographic trend has significant implications for long‐term care settings. A previous German study indicated that older adults, particularly those of advanced age, frequently experienced restricted living environments. The study further revealed a correlation between limited living space and increased care dependency (Buss et al. 2017). This association is especially pronounced among elderly individuals with dementia who reside in institutional long‐term care facilities, as these individuals constitute a high‐risk population for both mobility impairments and safety concerns. Moreover, such functional limitations have been shown to adversely affect both life expectancy and quality of life in advanced age (Schäufele et al. 2012).

The progression of dementia is also associated with a decline in specific motor abilities. Findings from a recent German study revealed that individuals with more severe dementia exhibit significant reductions in physical function (Schumacher et al. 2023). Impaired gait is particularly prevalent, rendering individuals with dementia particularly susceptible to falls. An international study reported that approximately 55% of individuals with dementia experience at least one fall annually, compared with prevalence rates of 25% among cognitively healthy individuals and 31% among those with mild cognitive impairment (Allali et al. 2017). Consequently, interventions aimed at fall prevention—including exercise, nutritional support and medication management—are considered essential components of care for this population (Allali et al. 2017).

## 2 Background

The impairments associated with dementia have a demonstrably adverse effect on life‐space mobility, meaning how freely and independently a person can get around in their daily life. A previous Brazilian study indicated that older adults diagnosed with Alzheimer's disease exhibit significantly reduced mobility compared with their cognitively healthy counterparts. Notably, 21% of individuals with Alzheimer's dementia were found to be homebound without external assistance, underscoring the need to focus on increasing mobility in this population (Langelli et al. 2023).

Further research has examined life‐space mobility within institutional long‐term care settings. One study reported that the majority of residents with dementia maintain a stable level of mobility within their living units. These findings suggested that superior physical performance and lower levels of apathy are positively correlated with an expanded life space (Sverdrup et al. 2021). Similarly, a German cross‐sectional study reported that life‐space mobility is markedly restricted among older, multimorbid individuals, particularly those with mild to moderate cognitive impairment. The study identified personal support and the provision of assistive equipment as critical determinants of life‐space mobility (Ullrich et al. 2019).

Individuals with dementia are highly dependent on professional caregiving for the maintenance of their mobility. Accordingly, targeted nursing interventions are essential to preserve and promote life‐space mobility in this population. A recent Dutch review critically examined the consequences of restrictive measures on the life space of individuals with dementia residing in care facilities with an eye towards human rights, ethics and gender‐specific dimensions. The review highlighted the need to systematically reflect upon and evaluate both restrictive and facilitative interventions influencing mobility. Furthermore, the review advocated for a paradigm shift in care practices, emphasising the development of individualised structures and strategies to enhance residents' quality of life (Sturge et al. 2024).

Restrictive measures, such as physical, chemical, or mechanical restraints that limit a person's movement or freedom, have widely recognised detrimental effects. These measures continue to be routinely employed in institutional long‐term care. Recent data indicate that 6.9% of residents in such facilities are subjected to restrictive interventions (Ritzi 2023). Of these, 5.2% were associated with deficiencies—such as inadequately padded restraint systems—that posed significant risks to the individuals concerned. Moreover, 3.5% of cases resulted in specific adverse outcomes, including the development of pressure ulcers attributable to restrictive measures (Ritzi 2023).

## 3 The Study

The current literature underscores the critical importance of investigating life–space mobility among individuals with dementia. Notably, the impact of liberty deprivation measures on life‐space mobility should be further examined. In the German context, research in this domain remains limited, as previous studies were predominantly cross‐sectional in nature or were conducted in healthcare systems that differ substantially from the structural and regulatory characteristics of the German healthcare system. Consequently, there is a pronounced need for robust longitudinal research focusing on individuals with dementia residing in long‐term inpatient care settings, as a cross‐sectional study would overlook important developments, correlations, and measures for people with dementia.

## 4 Objectives

The primary objective of the present study was to longitudinally assess the relationship between dementia and life‐space mobility among residents of long‐term inpatient care facilities, while additionally aiming to compare life‐space mobility between residents with and without dementia. The secondary objective is to examine the association between liberty deprivation measures and mobility among individuals with dementia.

Achieving the primary objective provides new insights into the impact of dementia on mobility in their living environments. This contributes to more targeted improvements in the care and quality of life for individuals with dementia in residential facilities. Furthermore, addressing the secondary objective offers valuable insights into the relationship between restrictive measures and mobility, highlighting the potential negative effects of such interventions on the freedom of movement and well‐being of those affected.

## 5 Methodology

### 5.1 Study Design

This quantitative, longitudinal study was designed to evaluate the trajectories of life‐space mobility among residents with and without dementia. Data on residents' life‐space mobility were collected over a 6‐month observation period.

### 5.2 Recruitment and Sampling

Data collection was conducted at three time points: baseline (t0), 3 months (t1) and 6 months (t2). The intervals between data collection points were consistently 3 months. The entire study period spanned from October 2023 to early July 2024. Baseline data (t0) were collected in November 2023 across five long‐term care facilities located in a northeastern region of Germany. The second wave of data collection (t1) occurred from mid‐January to mid‐February 2024, although one facility was surveyed in early April 2024. The final data collection (t2) was conducted in four facilities from early May to late May 2024, with one facility being surveyed in early July 2024.

### 5.3 Inclusion and Exclusion Criteria

Long‐term care facilities were initially contacted via mail and invited to participate in the study. An informational session was held to explain the study objectives and procedures, thereby facilitating the recruitment of residents. The participating facilities were offered incentives, such as staff training or monetary compensation. Resident recruitment was carried out by trained nursing staff within the participating facilities. The inclusion criteria were residents living in the participating long‐term care facilities at the time of data collection who provided informed consent. For residents who, due to their health condition, were no longer able to provide informed consent to participate in the study themselves, consent was obtained from their legal guardian. The exclusion criteria included short‐term care residents, individuals in the terminal phase of illness, and residents under 18 years of age.

### 5.4 Data Collection

After obtaining informed consent, the nursing staff conducted an individualised assessment of each resident's life‐space mobility. Life‐space mobility was operationalised as the extent of the resident's movement within and beyond the care facility. For this purpose, one dimension of the ‘Heidelberg Instrument for Assessing the Quality of Life of People with Dementia (H.I.L.D.E.)’, a validated tool specifically developed for evaluating quality of life in individuals with dementia, was employed (Becker et al. 2011). The range of movement captured by the H.I.L.D.E. instrument represents a subjectively significant environmental factor that holds individual meaning for residents and can therefore influence their quality of life. The assessment categorised the range of movement according to the following domains: (1) the resident's private room, (2) the immediate living area, (3) other locations within the facility (e.g., cafeteria, communal areas), (4) areas outside the facility but within its grounds (e.g., garden, terrace), and (5) locations outside the facility's premises (e.g., town, park) (Becker et al. 2011). Life‐space mobility, as measured by the H.I.L.D.E. instrument, was assessed by a responsible member of the nursing staff. Data regarding liberty deprivation were obtained from nursing documentation to determine its application among residents with and without dementia in long‐term care facilities. These interventions involve limiting the freedom of movement of care‐dependent individuals against their will. They range from less intrusive actions, such as engaging wheelchair brakes, to more severe forms like physical restraints. In German nursing homes, up to 10% of residents are subjected to such physical restraints on a daily basis. These measures are generally applied not with malicious intent but to prevent falls or due to caregiver overwhelm (Bavarian State Ministry of Health, n.d.). The study group comprised exclusively residents with a dementia‐related illness. The residents in question with a dementia‐related illness are individuals who have a medically confirmed diagnosis. The comparison group consisted of residents without a documented medical diagnosis of dementia.

### 5.5 Ethical Considerations

This study received approval with respect to ethics and data protection before the start of the study (Reg. No. BB 165/23).

### 5.6 Statistical Analyses

The demographic and clinical characteristics of all participants were documented at each data collection point and are presented as absolute and relative frequencies. To provide a comprehensive description of the sample, the participants were characterised by age and sex. In addition, the mean and standard deviation of age were reported separately for participants with and without dementia. Group differences in age were assessed using independent samples *t* tests, whereas differences in sex distribution were evaluated using the chi‐square test. Temporal changes in life‐space mobility were depicted using frequency distributions at each assessment point. Associations between dementia status (dementia vs. nondementia) and life‐space mobility were analysed using the chi‐square test. Life space mobility for residents with and without dementia at all three assessment times (t0, t1, t2) is expressed as frequencies and percentages for each range of movement category (Table 2). Additionally, the relative frequencies of liberty deprivation measures were reported for both individuals with and without dementia. The presence of measures restricting freedom was assessed using yes/no questions. The relationship between dementia diagnosis and liberty deprivation measures was examined using the chi‐square test and quantified with the effect size Cramer's *V*, with a 95% confidence interval (CI). The fisher's exact test was also employed at each time point to assess the association between life‐space mobility and liberty deprivation measures within both groups. The effect size was quantified using Cramer's *V* with a 95% confidence interval (CI). All the statistical analyses were conducted at a significance level of *α* = 0.05. Data analysis was performed using SPSS software version 29 (IBM Corp. 2023).

## 6 Results

### 6.1 Participant Characteristics

At baseline, the sample comprised 336 residents, 33.9% (*n* = 114) of whom had a confirmed diagnosis of dementia and 66.1% (*n* = 222) of whom had no confirmed diagnosis of dementia (Table 1). The sample at the first follow‐up (t1) comprised a total of 290 residents, thus indicating a dropout rate of 13.7% (*n* = 46) compared with baseline. Among the individuals who dropped out, 7.9% (*n* = 9) had dementia, and 16.7% (*n* = 37) did not have dementia. At the second follow‐up (t2), the sample consisted of 265 residents, thus indicating a dropout rate of 8.6% compared with t1. None of the individuals who dropped out between t1 and t2 had dementia, as all the dropouts were in the nondementia group (8.1%, *n* = 15). During the 6‐month observation period, the attrition rate was 21%.

**TABLE 1:** Sample characteristics (long‐term care residents) by time point, dementia diagnosis, age and gender.

Time point		t0 (*N* = 336)		t1 (*N* = 290)		t2 (*N* = 265)	
Diagnosis		Dementia, *n* (%)	No dementia, *n* (%)	Dementia, *n* (%)	No dementia, *n* (%)	Dementia, *n* (%)	No dementia, *n* (%)
Total		114 (33.9)	222 (66.1)	105 (36.2)	185 (63.8)	105 (39.6)	160 (60.4)
Dropout		—	—	9 (7.9)	37 (16.7)	0	15 (8.1)
Age	≤ 65	3 (0.9)	22 (6.5)	4 (1.4)	19 (6.6)	4 (2.6)	18 (6.8)
	> 65 ≤ 85	40 (11.9)	83 (24.7)	44 (15.2)	73 (25.2)	40 (15.19)	67 (25.3)
	> 85	71 (21.1)	117 (34.8)	56 (19.3)	92 (31.7)	61 (23)	74 (28)
	Missing	—		2 (0.7)		1 (0.4)	
	*M*	85.9	82.6	84.6	82.5	85.6	81.8
	SD	7.9	10.7	8.3	10.8	8.1	10.7
	*t*‐value	*p* = 0.002		*p* = 0.046		*p* < 0.001	
Sex	Male	30 (8.9)	76 (22.6)	27 (9.3)	75 (25.9)	26 (10)	52 (19.6)
	Female	83 (24.7)	146 (43.5)	55 (19)	127 (43.8)	79 (29.8)	105 (39.6)
	Missing	1 (0.3)		6 (2.1)		3 (1.1)	
	Male (total)	106 (31.5)		102 (35.2)		78 (29.4)	
	Female (total)	229 (68.2)		182 (62.8)		184 (63.5)	

*Note:* Discrepancies may be due to rounding errors.

Abbreviations: *M*, mean; SD, standard deviation.

### 6.2 Age

At baseline (t0), 0.9% (*n* = 3) of the residents with dementia were under 65 years of age, 11.9% (*n* = 40) were between 65 and 85 years of age, and 21.1% (*n* = 71) were over 85 years of age. A total of 6.5% (*n* = 22) of the residents without dementia were under 65 years, 24.7% (*n* = 83) were between 65 and 85 years of age, and 34.8% (*n* = 117) were over 85 years of age. At time t1, 1.4% (*n* = 4) of the residents with dementia were under 65 years of age, 15.2% (*n* = 44) were between 65 and 85 years of age, and 19.3% (*n* = 56) were over 85 years of age. A total of 6.6% (*n* = 19) of the residents without dementia were under 65 years of age, 25.2% (*n* = 73) were between 65 and 85 years of age, and 31.7% (*n* = 92) were over 85 years of age. At time t2, 2.6% (*n* = 4) of the residents with dementia were under 65 years of age, 15.1% (*n* = 40) were between 65 and 85 years of age, and 23% (*n* = 61) were over 85 years of age. A total of 6.8% (*n* = 18) of the residents without dementia were under 65 years of age, 25.3% (*n* = 67) were between 65 and 85 years of age, and 28% (*n* = 74) were over 85 years of age.

To investigate the potential relationship between age and dementia, an independent samples *t* test was performed for each assessment point (Table 1). To test that individuals with dementia would be older, a one‐tailed significance test was performed. The one‐sided *p* value was significant at t0 (*p* = 0.002), t1 (*p* = 0.046) and t2 (*p* < 0.001). The significance of the calculated *t*‐values supports our previously formulated hypothesis that residents with dementia are, on average, older than residents without dementia. Furthermore, the means and standard deviations of age were calculated for residents with and without dementia. At baseline (t0), the residents with dementia had a mean age of *M* = 85.9 years (SD = 7.9), while the residents without dementia had a mean age of *M* = 82.6 years (SD = 10.7). At t1, the mean ages for those with and without dementia were 84.6 years (SD = 8.3) and 82.5 years (SD = 10.8), respectively. At t2, the mean ages for those with and without dementia were 85.6 years (SD = 8.1) and 81.8 years (SD = 10.7), respectively. Overall, people with dementia were significantly older than people without dementia at all three time points.

### 6.3 Sex

At baseline (t0), the proportions of males and females were 31% (*n* = 106) and 68.2% (*n* = 229), respectively. Among the male residents, 8.9% (*n* = 30) had a dementia‐related illness, whereas this proportion was markedly higher among the female residents (24.7%, *n* = 83). Overall, 22.6% (*n* = 76) of the male participants and 43.5% (*n* = 146) of the female participants were in the nondementia group. At t1, the proportions of males and females were 35.2% (*n* = 102) and 62.8% (*n* = 182), respectively. Among the male residents, 9.3% (*n* = 27) had dementia‐related illnesses, whereas 19% (*n* = 55) of the female residents had dementia‐related illnesses. Overall, 25.9% (*n* = 75) of the male participants and 43.8% (*n* = 127) of the female participants were in the nondementia group. At t2, the proportions of males and females were 29.4% (*n* = 78) and 63.5% (*n* = 184), respectively. Among the male residents, 10% (*n* = 26) had a dementia‐related illness, whereas this proportion was almost three times higher among the female residents (29.8%, *n* = 55). Overall, 19.6% (*n* = 52) of the male participants and 39.6% (*n* = 105) of the female participants were in the nondementia group.

A chi‐square test was performed to investigate the correlation between sex and dementia (Table 1). The *p* values at all three time points were nonsignificant: t0 (*χ* ^2^(1) = 2.05, *p* = 0.153), t1 (*χ* ^2^(1) = 0.45, *p* = 0.504) and t2 (*χ* ^2^(1) = 2.10, *p* = 0.147). Therefore, there was no significant correlation between sex and dementia in this sample.

### 6.4 Living Space Mobility and Dementia

The results of the study showed relative frequencies of Living Space Mobility (Area of Movement) and Dementia, stratified by Time Point and Dementia Diagnosis (Table 2). If information on living space mobility was not available during data collection, it was recorded as missing data.

**TABLE 2:** Living space mobility (area of movement) and dementia, stratified by time point and dementia diagnosis.

Time point	t0		t1		t2	
Diagnosis	Dementia, *n* (%)	No dementia, *n* (%)	Dementia, *n* (%)	No dementia, *n* (%)	Dementia, *n* (%)	No dementia, *n* (%)
Resident's room	7 (6.1)	20 (9)	6 (5.7)	8 (4.3)	9 (8.6)	14 (8.8)
Residential area	53 (46.5)	58 (26.1)	44 (41.9)	51 (27.6)	52 (49.5)	47 (29.4)
Within the facility	13 (11.4)	31 (14)	17 (16.2)	44 (23.8)	15 (14.3)	27 (16.9)
Outside area of the facility	7 (6.1)	31 (14)	8 (7.6)	39 (21.1)	13 (12.4)	21 (13.1)
Outside the facility	17 (14.9)	62 (27.9)	12 (11.4)	34 (18.4)	14 (13.3)	50 (31.3)
Total	97 (85.1)	202 (91)	87 (82.9)	176 (95.1)	103 (98.1)	159 (99.4)
Missing	17 (14.9)	20 (9)	18 (17.1)	9 (4.9)	2 (1.9)	1 (0.6)
Chi‐square^a^ (*p*)	20.3 (*p* < 0.001)		15.4 (*p* = 0.004)		15.7 (*p* = 0.004)	

*Note:* Discrepancies may be due to rounding errors.

^a^ Degrees of freedom (df = 4).

At baseline, 114 people had a dementia‐related illness. The prevalence of living space mobility was 85.1% (*n* = 97). A total of 6.1% (*n* = 7) of the 114 residents with dementia only spent time in their room. A total of 46.5% (*n* = 53) of the residents with dementia spent time in the living area, and 11.4% (*n* = 13) of the residents with dementia spent time in the inpatient long‐term care facility. A total of 6.1% (*n* = 7) of residents with dementia spent time in the outside areas of the long‐term care facility. A total of 14.9% (*n* = 17) spent time away from the grounds of the long‐term care facility. In the nondementia group (*n* = 222), the prevalence of living space mobility was 91% (*n* = 202). Only 9% (*n* = 20) of the 222 residents without dementia only spent time in their room. A total of 26.1% (*n* = 58) of the residents without dementia were able to spend time in the living area, and 14% (*n* = 31) of the residents without dementia were able to spend time in places within the inpatient long‐term care facility. A total of 14% (*n* = 31) of the residents without dementia spent time in the outside areas of the inpatient long‐term care facility, and 27.9% (*n* = 62) of the residents without dementia spent time away from the grounds of the long‐term care facility.

At t1, 105 people had dementia‐related illnesses. The prevalence of living space mobility was 82.9% (*n* = 87). A total of 5.7% (*n* = 6) of the 105 residents with dementia only spent time in their room. A total of 41.9% (*n* = 44) of the residents with dementia spent time in the living area, and 16.2% (*n* = 17) of the residents with dementia spent time in the inpatient long‐term care facility. A total of 7.6% (*n* = 8) of residents with dementia spent time in the outside areas of the long‐term care facility. A total of 11.4% (*n* = 12) spent time away from the grounds of the facility. In the nondementia group (*n* = 185), the prevalence of living space mobility was 95.1% (*n* = 176). A total of 4.3% (*n* = 51) of the residents without dementia only spent time in their room. A total of 27.6% (*n* = 51) of the residents without dementia were able to spend time in the living area, and 23.8% (*n* = 44) of the residents without dementia spent time in the inpatient long‐term care facility. A total of 21.1% (*n* = 39) of the residents without dementia spent time in the outside areas of the long‐term care facility. A total of 18.4% (*n* = 34) of the residents without dementia spent time away from the grounds of the facility.

At t2, 105 people had dementia. The prevalence of living space mobility was 98.1% (*n* = 103). A total of 8.6% (*n* = 9) of the 105 residents with dementia only spent time in their room. A total of 49.5% (*n* = 52) of the residents with dementia spent time in the living area, and 14.3% (*n* = 15) spent time in the inpatient long‐term care facility. A total of 12.4% (*n* = 13) of the residents with dementia spent time in the outside areas of the long‐term care facility. A total of 13.3% (*n* = 14) of the residents with dementia spent time away from the grounds of the facility. In the nondementia group (*n* = 160), the prevalence of living space mobility was 99.4% (*n* = 159). A total of 8.8% (*n* = 14) of the residents without dementia only spent time in their room. A total of 29.4% (*n* = 47) of the residents without dementia spent time in the living area, and 16.9% (*n* = 27) of the residents without dementia were able to spend time in the inpatient long‐term care facility. A total of 13.1% (*n* = 21) of the residents without dementia spent time in the outside areas of the inpatient long‐term care facility. A total of 31.3% (*n* = 50) of the residents without dementia spent time away from the grounds of the facility. A chi‐square test was carried out to investigate the correlation between living space mobility and dementia, and the results revealed that there was a significant correlation between living space mobility and dementia at all three time points: t0 (*p* < 0.001), t1 (*p* = 0.004), t2 (*p* = 0.004).

### 6.5 Liberty Deprivation Measures and Dementia

At baseline, 10.5% (*n* = 12) of the residents with dementia were subjected to liberty deprivation measures. In comparison, 84.2% (*n* = 96) were not subjected to any liberty deprivation measures (Table 3). No data were available for 5.3% of these residents (*n* = 6). Among the residents without dementia, 10.8% (*n* = 24) were subjected to liberty deprivation measures; in contrast, 82.4% (*n* = 183) were not subjected to liberty deprivation measures. No data were available for 6.8% of these residents (*n* = 15).

**TABLE 3:** Liberty‐depriving measures (LDM) and dementia presented as absolute and relative frequencies at all three time points (t0, t1, t2).

Time point	t0		t1		t2	
Diagnosis	Dementia, *n* (%)	No dementia, *n* (%)	Dementia, *n* (%)	No dementia, *n* (%)	Dementia, *n* (%)	No dementia, *n* (%)
LDM	12 (10.5)	24 (10.8)	8 (7.6)	16 (8.7)	8 (7.6)	9 (5.6)
No LDM	96 (84.2)	183 (82.4)	94 (89.5)	163 (88.1)	94 (89.5)	147 (91.9)
Missing	6 (5.3)	15 (6.8)	3 (2.9)	6 (3.2)	3 (2.9)	4 (2.5)
Chi‐square^a^ (*p*)	0.02 (0.898)		0.10 (0.752)		0.43 (0.512)	
Cramer's *V* [CI]	0.01 [−0.11; 0.1]		0.02 [−0.13; 0.1]		0.04 [−0.09; 0.16]	

*Note:* Discrepancies may be due to rounding errors.

Abbreviations: CI, confidence interval; LDM, liberty deprivation measures.

^a^ Degrees of freedom (df = 1).

At t1, 7.6% (*n* = 8) of the residents with dementia were subjected to liberty deprivation measures, and 89.5% (*n* = 94) were not subjected to liberty deprivation measures. No data were available for 2.9% (*n* = 3) of the residents with dementia. Among residents without dementia, 8.7% (*n* = 16) were subjected to liberty deprivation measures, and 88.1% (*n* = 163) were not subjected to liberty deprivation measures. No data were available for 3.24% (*n* = 6) of these residents.

At t2, 7.6% (*n* = 8) of the residents with dementia were subjected to liberty deprivation measures, and 89.5% (*n* = 94) were not subjected to liberty deprivation measures. No data were available for 2.9% (*n* = 3) of the residents with dementia. A total of 5.6% (*n* = 9) of the residents without dementia were subjected to liberty deprivation measures, and 91.9% (*n* = 147) were not subjected to liberty deprivation measures. No data were available for 2.5% (*n* = 4) of the residents without dementia.

A chi‐square test was performed to investigate the correlation between liberty deprivation measures and dementia (Table 3); the results revealed that there was no significant correlation between liberty deprivation measures and dementia at any of the time points: t0 (*χ* ^2^(1) = 0.02, *p* = 0.898), t1 (*χ* ^2^(1) = 0.10, *p* = 0.752), and t2 (*χ* ^2^(1) = 0.43, *p* = 0.512); it can be assumed that there is no significant correlation between the liberty deprivation measures and dementia. These findings were confirmed by the very weak associations indicated by Cramer *V* values and the corresponding 95% CIs for each time point: t0 (*V* = 0.01 [−0.11; 0.1]), t1 (*V* = 0.02 [−0.13; 0.1]) and t2 (*V* = 0.04 [−0.09; 0.16]). These results show clearly that there are no correlations throughout the entire observation period. Therefore, there is no direct link between a person's dementia status and the use of liberty‐depriving measures, which is consistent over a 6‐month period.

### 6.6 Liberty Depriving Measures and Living Space Mobility

Furthermore, we investigated how liberty deprivation measures affect the living space mobility of long‐term care residents with and without dementia.

At baseline, a total of 25% of the residents with dementia and liberty deprivation measures only spent time in their room; 16.7% spent time in the living area; 16.7% spent time inside the care facility; 8.3% spent time in the outside areas of the facility; and 8.3% spent time away from the grounds of the facility (Table 4). A total of 25% of the residents without dementia and liberty deprivation measures only spent time in their room; 16.7% spent time in the living area; 4.2% spent time inside the facility; 16.7% spent time in the outside areas of the facility; and 20.8% spent time away from the grounds of the.

**TABLE 4:** Liberty deprivation measures and living space mobility in dementia, presented as percentages and frequencies.

Time point	t0		t1		t2	
Diagnosis	Dementia, *n* (%)	No dementia, *n* (%)	Dementia, *n* (%)	No dementia, *n* (%)	Dementia, *n* (%)	No dementia, *n* (%)
Resident's room	3 (25)	6 (25)	2 (25)	2 (12.5)	4 (50)	2 (22.2)
Residential area	2 (16.7)	4 (16.7)	0	5 (31.3)	2 (25)	2 (22.2)
Within the facility	2 (16.7)	1 (4.2)	2 (25)	0	2 (25)	4 (44.4)
Outside area of the facility	1 (8.3)	4 (16.7)	0	6 (37.5)	0	1 (11.1)
Outside the facility	1 (8.3)	5 (20.8)	2 (25)	0	0	0
Total	9 (75)	20 (83.3)	6 (75)	13 (81.3)	8 (100)	9 (100)
Missing	3 (25)	4 (16.6)	2 (25)	3 (18.8)	0	0
Fisher's exact test (*p*)^a^	9.79 (*p* = 0.018)	8.45 (*p* = 0.059)	11.34 (*p* = 0.006)	15.27 (*p* = 0.001)	12.42 (*p* = 0.005)	5.55 (*p* = 0.019)
Cramer's *V* [CI]	0.35 [0.18; 0.66]	0.26 [0.11; 0.44]	0.38 [0.26; 0.69]	0.31 [0.23; 0.52]	0.45 [0.21; 0.75]	0.25 [0.17; 0.45]

*Note:* Discrepancies may be due to rounding errors.

Abbreviations: CI, confidence interval; LDM, liberty deprivation measures.

^a^ Degrees of freedom (df = 4).

At t1, a total of 25% of the residents with dementia and liberty deprivation measures only spent time in their room; 0% spent time in the living area; 25% spent time inside the care facility; 0% spent time in the outside areas of the facility; and 25% spent time away from the grounds of the facility. A total of 12.5% of the residents without dementia and liberty deprivation measures only spent time in their room; 31.3% spent time in the living area; 0% spent time inside the facility; 37.5% spent time in the outside areas of the facility; and 0% spent time away from the grounds of the facility.

At t2, a total of 50% of the residents with dementia and liberty deprivation measures only spent time in their room; 25% spent time in the living area; 25% spent time inside the care facility; and 0% spent time in the outside areas of the facility or away from the grounds of the facility. A total of 22.2% of the residents without dementia and liberty deprivation measures only spent time in their room; 22.2% spent time in the living area; 44.4% spent time inside the facility; 11.1% spent time in the outside areas of the facility; and 0% spent time away from the grounds of the facility.

A fisher's exact test was performed to investigate the correlation between liberty deprivation measures and living space mobility in dementia patients (Table 4). The results revealed that there was a significant correlation between liberty deprivation measures and living space mobility in people with dementia (t0 *p* = 0.018; t1 *p* = 0.006; t2 *p* = 0.005). Similarly, among people without dementia (t0 *p* = 0.059; t1 *p* = 0.001; t2 *p* = 0.019), significant correlations between liberty deprivation measures and living space mobility was found at t1 and t2, whereas no significant association was observed at t0. Hence, it can be assumed that there is a significant correlation between the freedom‐deprivation measures and living space mobility in people with and without dementia. Liberty depriving measures reduce the living space mobility of residents independent of their dementia status. These findings were confirmed by the moderate effects sizes of Cramer's *V* values and the corresponding 95% CIs for each time point among people with dementia: t0 (*V* = 0.35 [0.18; 0.66]); t1 (*V* = 0.38 [0.26; 0.69]); t2 (*V* = 0.45 [0.21; 0.75]). The wide intervals suggests some uncertainty but confirms the association is above weak. Also for people without dementia the results supported weak to moderate association as indicated by Cramer's V values and the corresponding 95% CIs for each time point: t0 (*V* = 0.26 [0.11, 0.44]); t1 (*V* = 0.31 [0.23, 0.52]); t2 (*V* = 0.25 [0.17, 0.45]). The intervals indicate some variability but support the conclusion of a meaningful association.

## 7 Discussion

The primary aim of this study was to investigate the impact of dementia on the mobility of residents in long‐term care facilities, with a comparative analysis between residents with and without a dementia diagnosis. The secondary aim investigated the impact of deprivation of liberty measures on the mobility of residents with and without dementia.

At baseline, the study cohort comprised 336 residents recruited from five inpatient long‐term care facilities. During the 6‐month observation period, the attrition rate was 21%, which is considered comparatively low within this demographic. Attrition was presumed to result predominantly from resident mortality, relocation, or hospitalisation; however, these specific causes of dropout were not systematically recorded.

Over the 6‐month observation period, individuals with dementia consistently demonstrated lower levels of life‐space mobility than did residents without dementia. These group differences persisted across all assessment points. Statistical analysis revealed a significant association between a diagnosis of dementia and restricted mobility within the living environment. These findings are consistent with results from a Brazilian study, which reported that older adults with Alzheimer's disease exhibited significantly reduced maximum and independent life‐space mobility relative to cognitively unimpaired individuals (Langelli et al. 2023).

The findings of this study indicated that residents with dementia predominantly remain within the residential areas of the care facility, whereas residents without dementia exhibit a broader range of movement and more frequently access locations outside both the residential unit and the facility itself. These observations are consistent with those of a German cross‐sectional study, which reported that older, multimorbid individuals with mild to moderate cognitive impairment exhibit more significantly restricted life–space mobility (Ullrich et al. 2019). A Norwegian study corroborated these findings, demonstrating that residents with severe dementia exhibited significantly greater restrictions in both mobility and life space than individuals with moderate dementia. Furthermore, the severity of cognitive impairment was found to be significantly associated with reduced mobility (Sverdrup et al. 2021).

The secondary objective of this study was to examine the association between the use of liberty deprivation measures and life‐space mobility among individuals with dementia. The findings indicated that there was no significant relationship between a diagnosis of dementia and the use of liberty deprivation measures. However, a significant association was identified between the use of liberty deprivation measures and restricted life‐space mobility, independent of dementia status. These results are supported by international studies, which have shown that restraints—such as physical restraints or fixations—are employed more frequently among older adults with cognitive impairments or preexisting neurological conditions (Huang et al. 2014; Pu et al. 2023).

The use of restraints is associated with reduced life‐space mobility. Analysis of movement patterns revealed that residents with dementia who were subjected to restraints predominantly remained within their immediate residential area. In contrast, residents without dementia, even those who are subjected to liberty deprivation measures, were more frequently observed outside their residential area. These findings prompt further inquiry into the factors that may enable some residents who are subjected to liberty deprivation measures to access areas beyond the facility. Potential explanations may include variations in the type and severity of liberty deprivation measures applied, as well as the involvement of accompanying family members or additional caregivers. The nature of liberty deprivation measures can range from less restrictive interventions, such as raised bed rails, to more restrictive measures involving physical restraints. It also remains unclear whether the application of liberty deprivation measures is based on the resident's personal request or is mandated by judicial order. Furthermore, the possibility that residents may leave the facility under the supervision of family members or caregivers could also contribute to these observed patterns in life‐space mobility.

In our sample, significantly more women than men participated at all three time points. This distribution was evident in both groups of people with dementia and those without dementia. Our study provides a realistic picture of the inpatient care situation, as women live to an average age of 83.0 years, which is longer than the average lifespan of men (78.2 years) (Destatis 2024a). Furthermore, 69.9% of residents in need of care in fully inpatient long‐term care facilities were female (Destatis 2024b). At each of the three time points, the mean age of residents with dementia was older than that of residents without dementia. Accordingly, on average, individuals in the dementia group were older than those in the nondementia group. This age difference is consistent with established epidemiological patterns, as the prevalence of dementia increases markedly with advancing age (German Alzheimer Society e.V. 2024).

According to a report by the German Institute of Medical Documentation and Information (DIMDI 2009), age is the strongest risk factor for the development of dementia. The prevalence of dementia in inpatient long‐term care facilities increases substantially with advancing age, and dementia represents the leading cause of care dependency among older adults. Consequently, nursing home residents with dementia are markedly older than residents without dementia (Rieckmann et al. 2009).

Notably, across all assessment points in our sample, the proportion of female residents with dementia exceeded that of male residents. This observation is consistent with national data from Germany, which indicate that approximately 60% of the 1.42 million individuals diagnosed with dementia are female (WIdO 2025).

## 8 Limitations and Strengths

This longitudinal study had several limitations. Data collection was conducted at three distinct time points over a 6‐month period. Nevertheless, overall attrition rates were minimised through diligent sample management and the strong engagement of participating long‐term care facilities. Data collection relied on documentation from nurses, which may have resulted in incomplete capture of life‐space mobility due to potential gaps or insufficiencies in the records. For data protection reasons, information was collected using paper‐based questionnaires. Despite the involvement of trained staff, potential sources of error during data collection and data entry cannot be entirely excluded. Attrition occurred over the course of the study, primarily due to resident mortality, hospitalisation, or relocation. These cases could not be systematically tracked or analysed owing to the pragmatic nature of the research design. The analysis of restraint measures was constrained by a limited number of documented cases, as such interventions were recorded for only a small subset of residents. Consequently, drawing robust conclusions regarding the relationship between dementia and the use of restraints would require a larger sample size and more comprehensive patient documentation. Furthermore, this study could not examine in detail the type and rationale for the application of liberty deprivation measures (e.g., court order vs. personal request). The operationalization of life‐space mobility may have introduced discrepancies between caregiver assessments and the actual mobility of residents, potentially resulting in nonreporting bias. Data collection was conducted at three distinct time points over a 6‐month period. Furthermore, no time‐dependent analysis was performed in this longitudinal study.

## 9 Recommendation for Further Research

Future research should include longitudinal studies with larger sample sizes to increase the generalizability and robustness of the findings. Additionally, alternative methodologies for assessing life–space mobility should be considered to improve measurement accuracy. Building upon the current results, further longitudinal investigations are planned within long‐term care facilities to examine the observed associations in greater detail.

## 10 Conclusion

This quantitative longitudinal study investigated life‐space mobility and the application of liberty depriving measures among residents with and without dementia in long‐term residential care settings. A key strength of this study is its longitudinal design, which is particularly significant given the relative lack of longitudinal research on these topics in the existing literature. This design enabled the collection of continuous data on life‐space mobility among long‐term care residents both with and without dementia. By conducting repeated measurements over time, the study enhances the validity of its findings and allows for a more refined evaluation of changes and needs. Consequently, care and mobility requirements can be identified earlier, facilitating better‐informed care planning in the future. For subsequent longitudinal studies, it is advisable to include thorough documentation and analysis of attrition factors—such as mortality and relocation—to improve the accuracy and interpretability of the results.

## Author Contributions

Nico Marcus Haller played a key role in the development and conception of the research project. He was also responsible for the development, collection, procurement and provision of the relevant data and sources. He also analysed, evaluated and interpreted the data and sources obtained and drew the corresponding conclusions. He also wrote the manuscript. Lena Knüppel was involved in the development, collection, procurement and provision of the data and the corresponding sources. She also actively supported the literature research and thus made a significant contribution to the success of the project. She helped shape the data analysis in the initial phase. Simone Freitag was significantly involved in the analysis, evaluation and interpretation of the data and sources and drew the corresponding conclusions. She also undertook the critical revision of the manuscript, particularly with regard to key content and methodological aspects. Lars Kaderali was involved in the analysis and evaluation of the data. He also provided statistical advice by selecting suitable methods for statistical evaluation and supporting their application. Steve Strupeit played a key role in the development and conception of the research project. He also analysed, evaluated and interpreted the data and sources collected and drew conclusions from them. He also supported the writing of the manuscript.

## Funding

The authors have nothing to report.

## Disclosure

Statistics: The authors confirm that the methods used in the data analyses were appropriately applied to their data within the context and design of their study, and that the statistical results were correctly implemented and interpreted. The statistics were reviewed prior to submission by Prof. Dr. Lars Kaderali, who is also listed as co‐author. The authors agree to take responsibility for ensuring that the choice of statistical approach is appropriate and that it has been correctly conducted and interpreted as a condition for submission to the journal.

## Conflicts of Interest

The authors declare no conflicts of interest.

## Data Availability

The data that support the findings of this study are available from the corresponding author upon reasonable request.
